# Giant Negative Magnetoresistance Effect in La_1−*x*_Pr*_x_*Fe_12_B_6_ (*x* = 0.05, 0.15) Compounds

**DOI:** 10.3390/ma19204419

**Published:** 2026-10-21

**Authors:** Zhipan Ma, Yiming Chen, Zhengwei Yang, Shan Dong

**Affiliations:** 1 Rocket Force University of Engineering, Xi’an 710025, China; 2 National Key Laboratory of Solid Rocket Propulsion, Rocket Force University of Engineering, Xi’an 710025, China; 3 MOE Key Laboratory of Material Physics and Chemistry Under Extraordinary Conditions, School of Physical Science and Technology, Northwestern Polytechnical University, Xi’an 710072, China

**Keywords:** LaFe_12_B_6_, magnetoresistance, itinerant-electron metamagnetism, kinetic arrest, magnetic materials

## Abstract

LaFe_12_B_6_ is a rare itinerant-electron metamagnet, and substitution of rare-earth elements can significantly influence its field-induced phase transition and magnetocaloric effects. However, how such elemental substitution modulates its magnetoelectrical transport properties remains unclear. Here, we report electrical transport measurements on La_1−*x*_Pr*_x_*Fe_12_B_6_ (*x* = 0.05, 0.15) compounds between 3 and 150 K in fields up to 90 kOe using the four-wire method on characterized polycrystals containing 9.5 and 3.8 wt.% Fe_2_B, respectively. Samples were thermally reset and demagnetized prior to each isothermal test. At 3 K, magnetoresistance (*MR*) reproduces the following magnetization features: two abrupt *MR* drops at 41 and 67 kOe for *x* = 0.05. Below the first critical field, the *MR* becomes positive (+20% for *x* = 0.05, +5% for *x* = 0.15) and then drops abruptly. The steps broaden and merge into a smooth response as the temperature rises. The largest negative *MR* values measured, −78% (*x* = 0.05, 40 K) and −68% (*x* = 0.15, 70 K), lie close to the corresponding magnetic transitions. The incomplete resistivity recovery observed after field removal—most pronounced at low temperature for both compositions and vanishing near 120 K—reveals a kinetically arrested transformation. By combining magneto-transport with magnetization measurements, the giant negative magnetoresistance (up to −78%) of La_1−*x*_Pr*_x_*Fe_12_B_6_ closely tracks the stepwise metamagnetic transition, via field-suppressed spin-disorder scattering, with the effect extending above *T*_C_ for the Pr-richer composition.

## 1. Introduction

The magnetoresistance (*MR*) effect probes how conduction electrons are scattered in a metal, and its magnitude varies over many orders between different families of materials [1,2]. An ordinary metal shows only a small positive *MR* arising from the Lorentz-force deflection of the electron trajectories; large effects require the field to modify the magnetic state itself, as in colossal-magnetoresistance manganites where charge order melts, in multilayers where spin-dependent scattering is switched by the relative alignment of ferromagnetic layers [3], and in semimetals showing extreme magnetoresistance [2]. An attractive route is therefore to exploit metamagnetic compounds in which a field converts an antiferromagnetic (AFM) ground state into a ferromagnetic (FM) one: these two states differ strongly in spin disorder, so the transformation changes the resistivity abruptly and simultaneously generates magnetocaloric and magnetostrictive responses [4,5,6,7].

LaFe_12_B_6_ is a metamagnet with a SrNi_12_B_6_-type structure [8], and being free of 4f electrons, it is an itinerant-electron system whose magnetism resides entirely on two inequivalent Fe sites [9,10,11]. Below about 8 K, an applied field drives a first-order AFM-FM transition that proceeds through ultrasharp, avalanche-like magnetization steps [12,13,14], and the same magneto-structural transition yields both normal and inverse magnetocaloric effects, with a maximum magnetic entropy change of about 19 J/(kg·K) at 7 T, together with giant-volume magnetostriction and an unusually strong sensitivity to hydrostatic pressure [15,16,17]. Isostructural RT12B6 compounds display related field-induced transitions [18,19,20,21,22,23]. In the isostructural Co-based series *RE*Co_12_B_6_ (*RE* = Ce, Pr, Nd), by contrast, the *RE* and Co moments align antiparallel to give a ferrimagnetic ground state that orders at 130–160 K, and the transition is governed by the spin-polarized Co-3d states with a second-order, 3D-Heisenberg character [24]. Applications of the Fe-based members are nevertheless hindered by critical fields of several tesla and pronounced hysteresis [15]. Substituting part of the La by another rare earth (*RE*) has proved the most effective way of tuning this balance: Ce substitution produces multiple magnetization plateaus and a coupled magnetic–crystallographic transition [25,26], Nd substitution stabilizes the ferromagnetic state [27], and early work has shown that as little as 5% Pr shifts the transition temperature and the critical field while preserving a large entropy change [28,29]. Quantitative magneto-transport measurements nevertheless remain scarce, the only systematic study so far being that of a Ce-substituted crystal, which revealed spontaneous resistivity jumps occurring at strictly constant temperature and field after a period of incubation [30]. In particular, the origin of the giant negative *MR*, the way in which this response is correlated with the individual steps of the metamagnetic process, and how the doping level governs both its magnitude and its temperature window have not been established.

In this work, we address that question through a systematic study of the electrical transport of La_0.95_Pr_0.05_Fe_12_B_6_ and La_0.85_Pr_0.15_Fe_12_B_6_, compositions selected from our earlier magnetic investigation because they combine low impurity content (9.5 and 3.8 wt.% Fe_2_B) with appreciably different transition temperatures [28]. Resistivity was measured by the standard four-wire method between 3 and 150 K in fields of up to 90 kOe. Because the first-order transition shows pronounced hysteresis and magnetic memory, each isotherm was preceded by demagnetization and heating above 150 K, so that every field sweep started from the same virgin AFM state. The transport data are analyzed jointly with the magnetization curves, allowing each resistivity feature to be assigned to a specific step of the metamagnetic process.

The present results establish how the giant negative *MR* of La_1*−x*_Pr*_x_*Fe_12_B_6_ is generated and how it is tuned by the Pr content. At 3 K, the resistivity drops abruptly at the critical fields of the magnetization steps (41 and 67 kOe for *x* = 0.05; 41 kOe for *x* = 0.15). And *MR*(*H*) reaches −78% at 40 K and −68% at 70 K. The temperature of maximum *MR* follows the Pr-induced shift of the transition temperature, which rises from 47 K for *x* = 0.05 to 63 K for *x* = 0.15 (0.5 T, zero-field cooling [28]), giving a composition knob to place the largest response at a chosen cryogenic point. An *MR* of −72% survives at 55 K, only about 8 K above the Curie temperature of La_0.95_Pr_0.05_Fe_12_B_6_, so the dominant scattering contrast is FM versus short-range-ordered paramagnetic, not AFM versus FM. Partial recovery below these thresholds and full recovery only by 120 K indicate a martensitic-like arrest.

## 2. Materials and Methods

La_1−*x*_Pr*_x_*Fe_12_B_6_ (*x =* 0.05, 0.15) was synthesized by the arc-melting method from high-purity La (99.9 wt.%), Pr (99.9 wt.%), Fe (99.9 wt.%) and B (99.99 wt.%) metals in an Ar gas atmosphere. A 2 wt.% addition of La was used to suppress the formation of an Fe_2_B impurity phase. The ingots obtained were finally annealed at 1173 K for 21 days and then rapidly quenched in cold water. The crystal structures of the samples were checked by powder XRD (SmartLab 9KW, Rigaku, Akishima, Tokyo, Japan). The crystal structure and purity of the samples were determined by refinement using the FullProf Suite (September 2020 version) [31].

**Figure 1:**
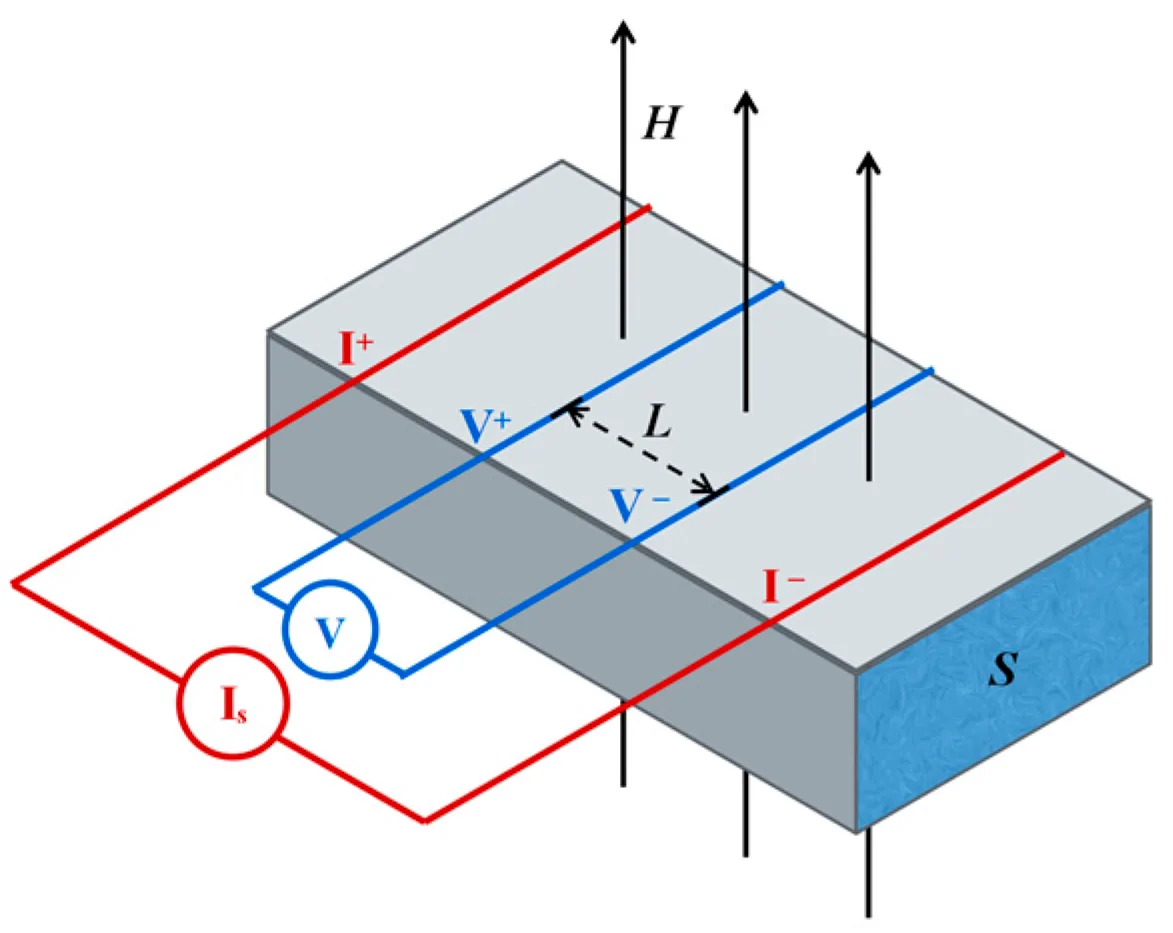
Schematic diagram of the longitudinal four-probe transport measurement. ***L*** is the distance between voltage leads, ***H*** is the applied magnetic field, and ***S*** is the cross-sectional area of the sample.

The magnetic data for La_1−*x*_Pr*_x_*Fe_12_B_6_ were collected using the Magnetic Property Measurement System (MPMS-7, Quantum Design, Inc., San Diego, CA, USA). Before each measurement of the variation in magnetization with magnetic field, the sample was cooled from a zero-magnetic field condition above 150 K to the target temperature. After the measurement was completed, the sample was demagnetized in the oscillate mode (the field alternately reversed between ±10 kOe, with its amplitude progressively reduced to zero at 300 Oe/s). For electrical property measurements, rectangular blocks (about 6 × 3 × 0.8 mm^3^) were cut from the ingots. After surface polishing, silver electrodes were deposited onto the samples via evaporation. The electrical transport properties of the materials under magnetic field were characterized using a Physical Property Measurement System (PPMS, 0–9 T, Quantum Design, Inc., San Diego, CA, USA) with the standard four-wire method. The schematic diagram of the measurement circuit is shown in Figure 1. The magnetic field direction is perpendicular to the test plane and the current flow direction. During the test, the sample was cooled from 150 K under zero magnetic field to the target temperature. After the temperature stabilized, the magnetic field was increased at a rate of 200 Oe/s, and resistivity measurements were taken at intervals of 1000 Oe. To prevent interference of the varying magnetic field with electrical signals, resistivity testing was performed after waiting for 30 s once the magnetic field reached its set value, and the test current was set to 1 mA. To erase the magnetic memory acquired during each field sweep, the sample was demagnetized in the oscillate mode of the PPMS—the field alternately reversed between ±10 kOe, with its amplitude progressively reduced to zero at 300 Oe/s, then warmed to 150 K in zero field and cooled to the next target temperature; every *MR* curve therefore started from the same virgin AFM state. The resistivity was calculated by

(1) $$\rho =\frac{V}{I_{S}}\frac{S}{L},$$

where ***L*** is the distance between the voltage poles and ***S*** is the cross-sectional area of the sample. The *MR* was calculated as

(2) $$MR=\frac{\rho (H,T)-\rho (0,\;T)}{\rho (0,T)},$$

*MR* calculations in this paper all use the data measured during the initial magnetic field increase.

## 3. Results

### 3.1. Structural Characterization and Zero-Field Resistivity

The Rietveld refinement parameters and impurity content of the La_1−*x*_Pr*_x_*Fe_12_B_6_ (*x =* 0.05, 0.15) compounds are shown in Table 1. The contents of the impurity phases (Fe_2_B) were approximately 9.5 and 3.8 wt.% for La_0.95_Pr_0.05_Fe_12_B_6_ and La_0.85_Pr_0.15_Fe_12_B_6_. The nominal compositions were confirmed by XRD Rietveld refinement.

**Figure 2:**
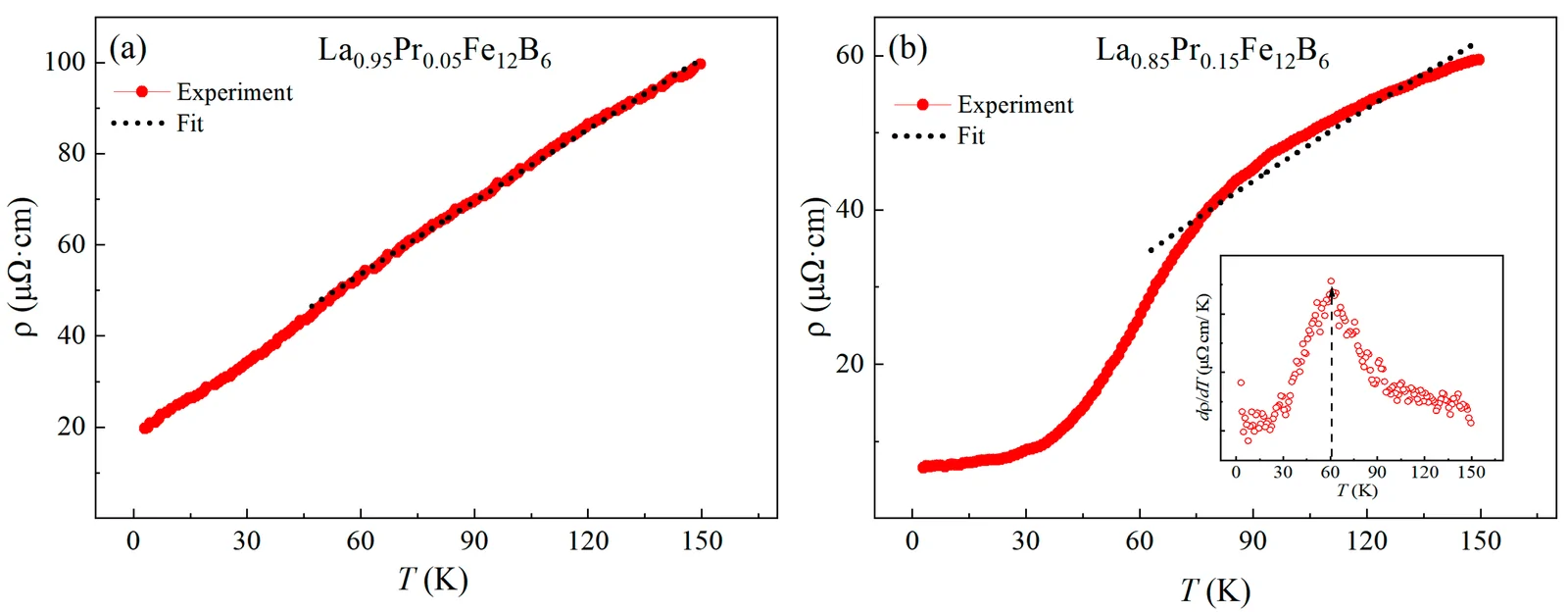
Resistivity versus temperature curves of La_1−*x*_Pr*_x_*Fe_12_B_6_. (**a**) La_0.95_Pr_0.05_Fe_12_B_6_; (**b**) La_0.85_Pr_0.15_Fe_12_B_6_. The inset shows the resistivity change rate vs. temperature curve of La_0.85_Pr_0.15_Fe_12_B_6_.

First, the resistivity properties of La_0.95_Pr_0.05_Fe_12_B_6_ and La_0.85_Pr_0.15_Fe_12_B_6_ were tested under zero magnetic field in the range of 3–150 K. Figure 2a shows the temperature dependence of resistivity for La_0.95_Pr_0.05_Fe_12_B_6_. The sample exhibits typical metallic behavior: the resistivity decreases linearly with decreasing temperature, which is basically consistent with the electrical transport test results of pure LaFe_12_B_6_ without an external magnetic field [9]. Figure 2b shows the temperature dependence of resistivity for La_0.85_Pr_0.15_Fe_12_B_6_ under zero magnetic field in the range of 3–150 K. The resistivity decreases gradually with decreasing temperature, which is significantly different from the result of La_0.95_Pr_0.05_Fe_12_B_6_ without an external magnetic field. The resistivity of La_0.95_Pr_0.05_Fe_12_B_6_ at 150 K is about 99.65 μΩ·cm, which is obviously larger than 59.45 μΩ·cm for La_0.85_Pr_0.15_Fe_12_B_6_. At 3K, the resistivity is 19.75 μΩ·cm and 6.59 μΩ·cm, respectively. The residual resistivity ratio (*RRR*) is calculated by *RRR* = *ρ*(150 K)/*ρ*(3 K). Compared with La_0.95_Pr_0.05_Fe_12_B_6_ (*RRR* ≈ 5.0), La_0.85_Pr_0.15_Fe_12_B_6_ shows a ~1.8-fold enhanced residual resistivity ratio (*RRR* ≈ 9.0). The elevated residual resistivity of La_0.95_Pr_0.05_Fe_12_B_6_ may arise from two sources: (i) interfacial scattering at its larger Fe_2_B fraction (9.5 vs. 3.8 wt.%) or (ii) scattering by point defects and the random occupation of the same crystallographic site by La and Pr. Together with the XRD refinement results of the two samples, the change in resistivity confirms the conclusion that the impurity content in La_0.85_Pr_0.15_Fe_12_B_6_ is lower than that in La_0.95_Pr_0.05_Fe_12_B_6_.

In addition, unlike the nearly linear resistivity change in Figure 2a, the resistivity temperature change rate in La_0.85_Pr_0.15_Fe_12_B_6_ has an obvious slope variation. By differentiating the curve in Figure 2b, the resistivity change rate vs. temperature curve of La_0.85_Pr_0.15_Fe_12_B_6_ was obtained. It can be seen that the resistivity change rate of the sample reaches its maximum at 61 K. Moreover, this temperature is very close to the Curie temperature (*T*_C_ = 63 K) of La_0.85_Pr_0.15_Fe_12_B_6_ measured at 5 kOe under zero-field cooling (ZFC), the corresponding value for La_0.95_Pr_0.05_Fe_12_B_6_ being 47 K under the same conditions [28], indicating that the resistivity change rate is closely related to the magnetic phase transition of the material, and the change in magnetic structure leads to a change in the conductivity of the material. To quantitatively assess the contribution of critical spin fluctuations to the charge transport, the resistivity above *T*_C_ was fitted to *ρ* = *ρ*_0_+ A*T*^n^ [32], with *ρ*_0_ fixed to the measured 3 K value to avoid parameter correlation. The fits yield effective exponents n = 0.95 for *x =* 0.05 and n = 0.79 for *x =* 0.15 (R^2^ = 0.998 and 0.955). The nearly linear *T* dependence for *x =* 0.05 is dominated by electron–phonon scattering, while the sublinear behavior of *x =* 0.15 indicates enhanced scattering by critical spin fluctuations approaching *T*_C_. n is an effective exponent for fluctuation contributions rather than a universal critical index, as the FM–PM transition of these itinerant-electron metamagnets is first-order.

### 3.2. Step-like Magnetoresistance and Its Temperature Evolution

In addition, magnetic measurements of La_1*−x*_Pr*_x_*Fe_12_B_6_ showed that the magnetization of both compositions jumps in a step-like manner with increasing magnetic field at extremely low temperatures. These magnetization changes are very similar to those observed in manganite colossal magnetoresistance materials, so the magnetoresistance (*MR*) of La_0.95_Pr_0.05_Fe_12_B_6_ and La_0.85_Pr_0.15_Fe_12_B_6_ was studied.

**Figure 3:**
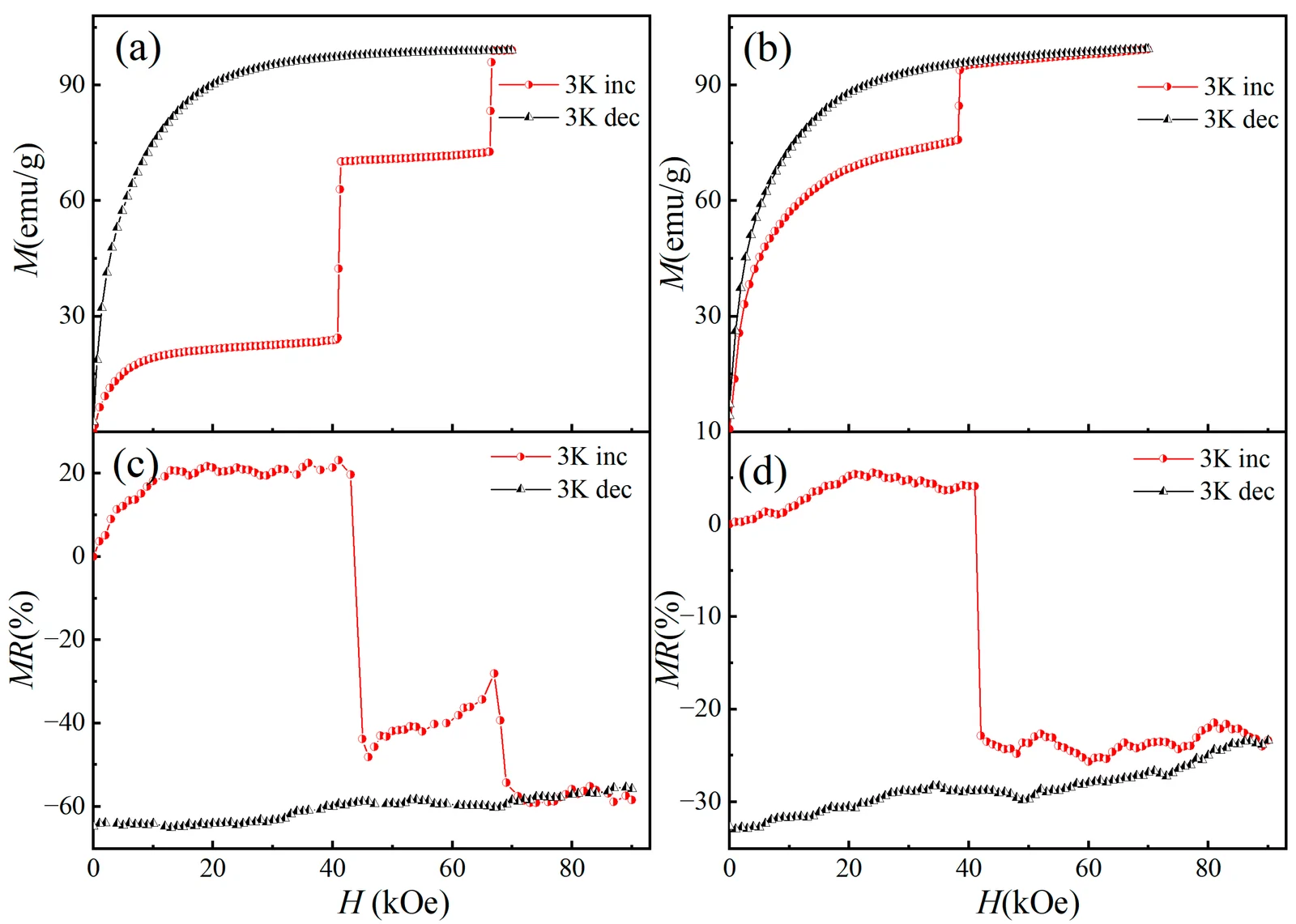
Magnetization and magnetoresistance versus magnetic field at 3 K for La_1−*x*_Pr*_x_*Fe_12_B_6_. (**a**,**c**) La_0.95_Pr_0.05_Fe_12_B_6_; (**b**,**d**) La_0.85_Pr_0.15_Fe_12_B_6_. “inc” and “dec” denote the increasing and decreasing magnetic field processes, respectively.

Figure 3 shows the *M*(*H*) and *MR*(*H*) curves of La_0.95_Pr_0.05_Fe_12_B_6_ (a,c) and La_0.85_Pr_0.15_Fe_12_B_6_ (b,d), where the red circles and black triangles denote the field-increasing (inc) and field-decreasing (dec) sweeps, respectively. For La_0.95_Pr_0.05_Fe_12_B_6_, Figure 3a shows that the magnetization exhibits two step-like jumps during the initial magnetization process and does not coincide with the demagnetization curve, while Figure 3c shows the corresponding *MR*(*H*). Below the first critical field (≈41 kOe), the resistivity increases gradually with increasing field and the *MR* reaches a positive value of about +20%; at 41 kOe, the *MR* drops abruptly from about +20% to about −45%. Between 41 and 67 kOe, the resistivity again increases gradually with field, and the second step occurs near 67 kOe, where the *MR* drops to about −59%. The black dec curve in Figure 3c lies below the inc curve over the whole field range and does not retrace the initial magnetization path, indicating pronounced irreversibility at 3 K. For La_0.85_Pr_0.15_Fe_12_B_6_, Figure 3b shows that the magnetization increases continuously up to about 41 kOe and then rises in a single step to saturation; the high-field step near 67 kOe observed in the *x =* 0.05 sample is absent. Accordingly, Figure 3d shows only one *MR* jump: below 41 kOe, the *MR* rises only slightly (by about 5%), and at 41 kOe, it drops abruptly to about −25%, remaining nearly constant at higher fields. The dec curve is again shifted downward relative to the inc curve, indicating that the field-induced FM state is not fully recovered upon field removal. The comparison between the two compounds shows that increasing the Pr content stabilizes the FM state and removes the intermediate magnetic phase: the two-step transformation of La_0.95_Pr_0.05_Fe_12_B_6_ simplifies to a single step in La_0.85_Pr_0.15_Fe_12_B_6_, and the positive *MR* preceding the transition weakens from about +20% (*x =* 0.05) to only a few percent (*x =* 0.15).

**Figure 4:**
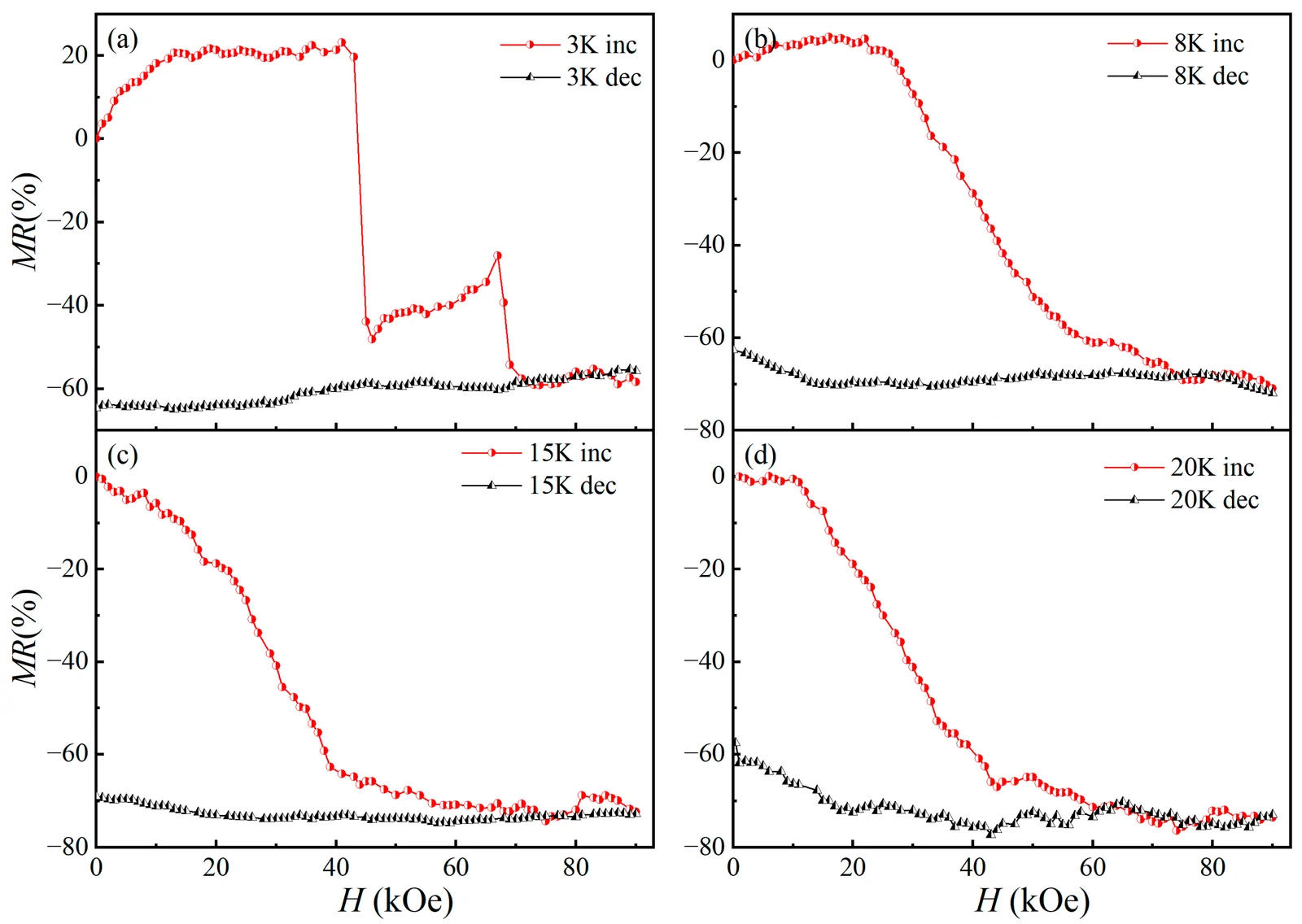
*MR*(*H*) curves of La_0.95_Pr_0.05_Fe_12_B_6_ at different temperatures. (**a**) 3 K; (**b**) 8 K; (**c**) 15 K; (**d**) 20 K.

Figure 4 shows the *MR*(*H*) curves of La_0.95_Pr_0.05_Fe_12_B_6_ at 3, 8, 15 and 20 K. The step-like resistivity change broadens above 8 K and evolves into a smooth response with reduced but still visible hysteresis. The resistivity curve during demagnetization does not overlap with that during magnetization, but the resistivity change during demagnetization is small. In addition, the maximum *MR* value of La_0.95_Pr_0.05_Fe_12_B_6_ increases gradually with increasing temperature: about −59% at 3 K, −72% at 8 K, −75% at 15 K and −74% at 20 K. The smearing of the steps is the direct consequence of the thermally assisted nucleation of the ferromagnetic phase: thermal fluctuations lower the effective barrier separating the AFM and FM free-energy minima, so that the avalanche-like growth of FM domains is gradually replaced by a continuous, thermally broadened transformation [12,13,14]. Already at 15 K, the maximum *MR* of La_0.95_Pr_0.05_Fe_12_B_6_ reaches −75%, and it keeps growing as the transition temperature is approached, the behavior expected for a transformation that becomes increasingly susceptible to the applied field.

**Figure 5:**
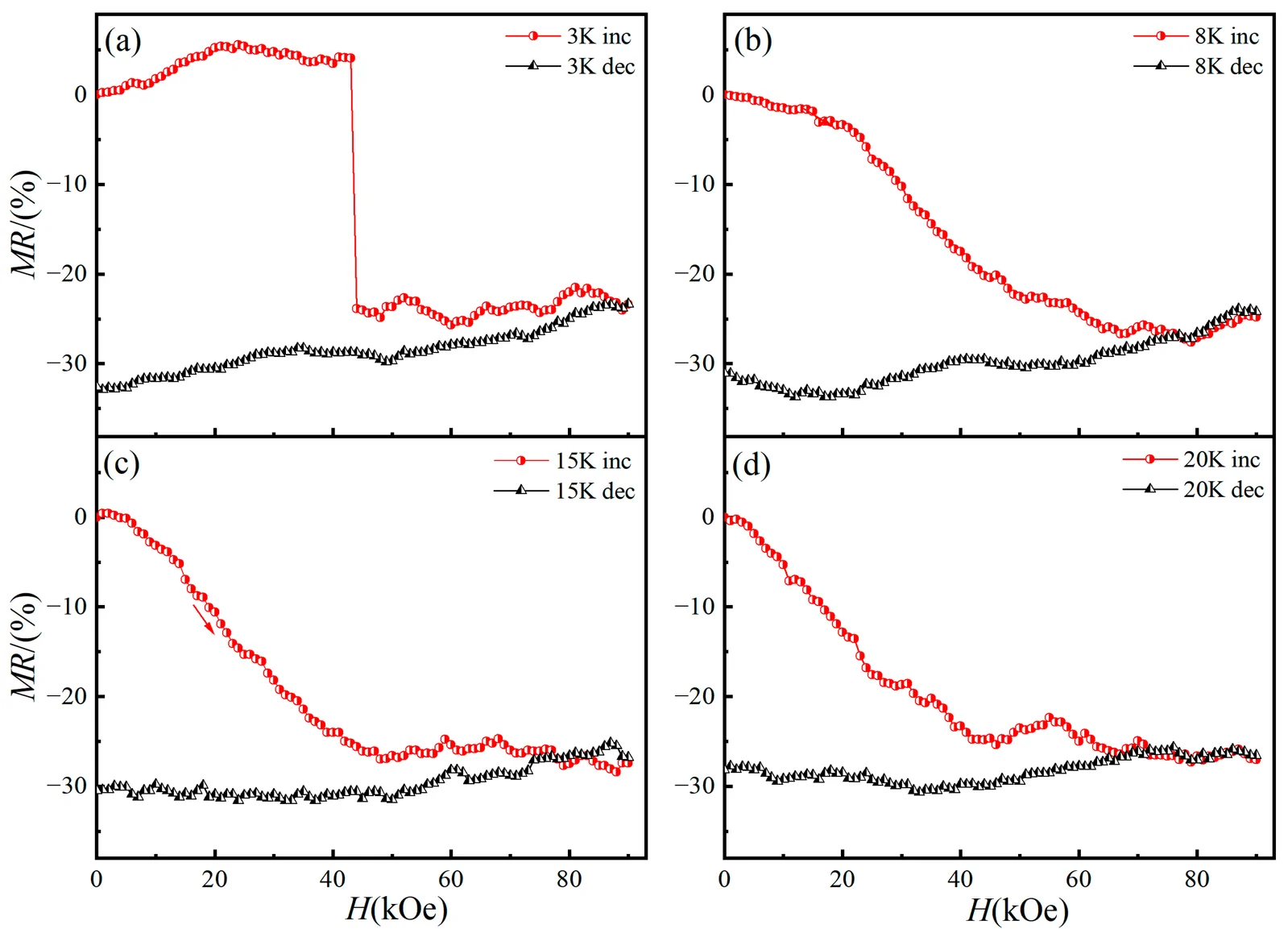
*MR*(*H*) curves of La_0.85_Pr_0.15_Fe_12_B_6_ at different temperatures. (**a**) 3 K; (**b**) 8 K; (**c**) 15 K; (**d**) 20 K.

Figure 5 shows the *MR*(*H*) curves of La_0.85_Pr_0.15_Fe_12_B_6_ at 3, 8, 15, and 20 K. The magnetization step at 41 kOe (Figure 3b) is mirrored by a step-like *MR* jump in Figure 5. Importantly, the resistivity decreases significantly during demagnetization, and this phenomenon disappears with increasing temperature. The maximum *MR* values calculated are −24% at 3 K, −27% at 8 K, −28% at 15 K, and −27% at 20 K, which are significantly smaller than those of La_0.95_Pr_0.05_Fe_12_B_6_ at the same temperatures. This composition dependence is consistent with the phase-fraction picture in which AFM, FM and paramagnetic phases coexist, and their relative concentrations, and hence the transport response, are governed by the magnetic and thermal history of the specimen [25,26]. Increasing the Pr content stabilizes the FM arrangement and reduces the AFM fraction available for the field-induced transformation, which naturally accounts for the lower *MR* ratio of La_0.85_Pr_0.15_Fe_12_B_6_. The finite remanent magnetoresistance observed after removal of the field at 3 K reflects the irreversibility of the low-temperature transformation: the virgin AFM state can only be restored by warming the specimen above *T*_C_ and cooling it again in zero field, the very protocol adopted in the present work (heating above 150 K followed by oscillating-field demagnetization).

### 3.3. Magnetoresistance near and Above the Curie Temperature

**Figure 6:**
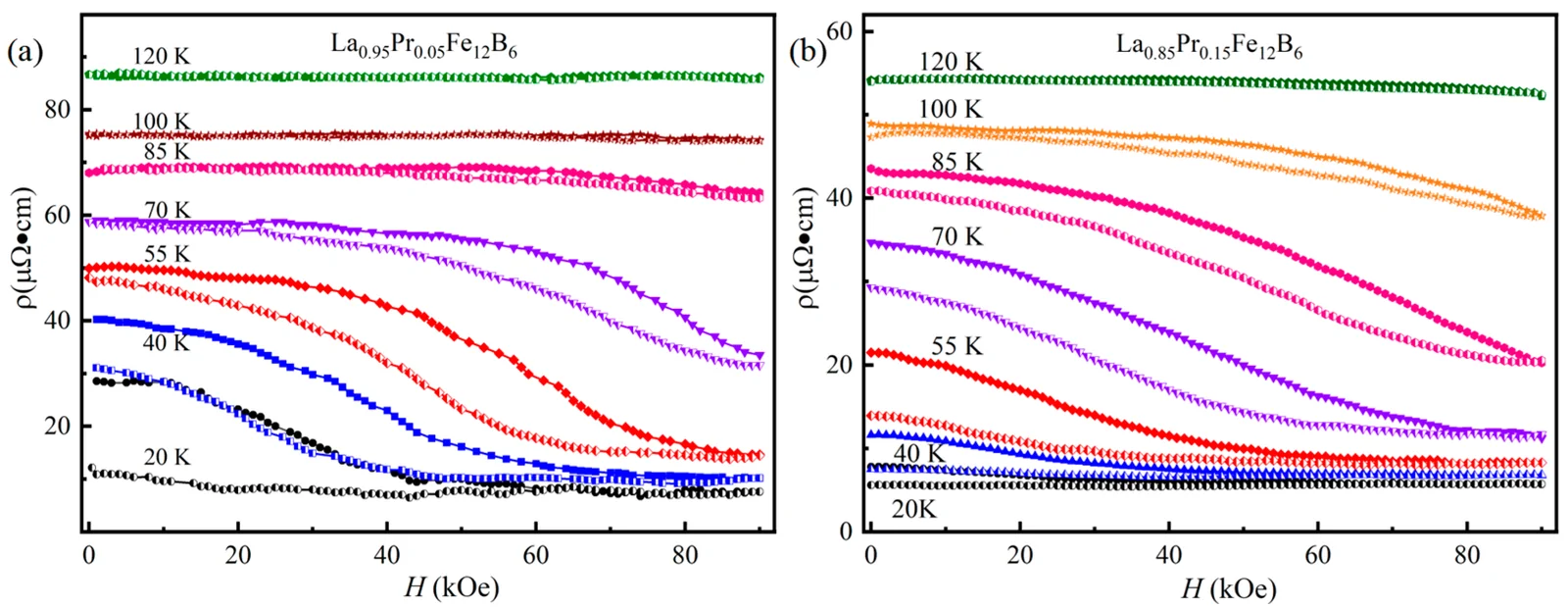
*ρ*(*H*) curves of La_0.95_Pr_0.05_Fe_12_B_6_ and La_0.85_Pr_0.15_Fe_12_B_6_ at different temperatures. (**a**) La_0.95_Pr_0.05_Fe_12_B_6_; (**b**) La_0.85_Pr_0.15_Fe_12_B_6_. Fully filled symbols: magnetic field up-sweep; half-filled symbols: magnetic field down-sweep.

Figure 6 shows the *ρ*(*H*) curves of La_0.95_Pr_0.05_Fe_12_B_6_ and La_0.85_Pr_0.15_Fe_12_B_6_ at different temperatures. The resistivity varies significantly with the magnetic field at different temperatures. The demagnetization curve at 20 K is similar to that in Figure 3, but the demagnetization curve at 40 K shows an obvious rise. At temperatures above 40 K, when the magnetic field decreases to 0, the resistivity is almost the same as the initial unmagnetized resistivity. The maximum *MR* values calculated from Figure 6a at 20 K and 40 K are −74% and −78%, respectively. Above *T*_C_, the resistivity at 55 K is still significantly affected by the magnetic field, with a maximum *MR* of −72%. As the temperature continues to rise, the influence of the magnetic field on resistivity gradually decreases: at 85 K, the resistivity hardly changes with the magnetic field, and the calculated *MR* is −7%; at 100 K, the *MR* is only about −1%.

For La_0.85_Pr_0.15_Fe_12_B_6_, the demagnetization curve at 20 K is similar to that in Figure 6a, but above 40 K, the resistivity rises gradually as the field is decreased and the remanent (unrecovered) resistivity persists up to about 85 K; only at 120 K does the demagnetization curve nearly overlap with the magnetization curve. The *MR* values calculated from Figure 6 increase gradually from 30 K to 70 K: the maximum *MR* values at 40 K, 55 K, and 70 K are −42%, −62%, and −68%, respectively. As the temperature continues to rise, the influence of the magnetic field on resistivity gradually decreases, but the resistivity of La_0.85_Pr_0.15_Fe_12_B_6_ still changes significantly with the magnetic field at 120 K, with a calculated *MR* of −4%.

The persistence of a sizable negative *MR* well above *T*_C_ (−72% at 55 K for *x =* 0.05) deserves particular commentary. A comparable effect has been reported for the Ce-substituted compound, where no perceptible resistivity anomaly is detected at the zero-field AFM-PM transition and a pronounced resistivity change appears only at the field-induced FM-PM transformation [25]. This indicates that the dominant scattering contrast in the present compounds is that between the field-aligned FM state and the disordered (or short-range-ordered) PM state, rather than that between the AFM and FM states: scattering of the conduction electrons by FM magnons and phonons is markedly weaker than in the AFM or PM arrangement, which is why the resistivity drops abruptly once the metamagnetic transition is completed and recovers only gradually as the field is removed.

**Figure 7:**
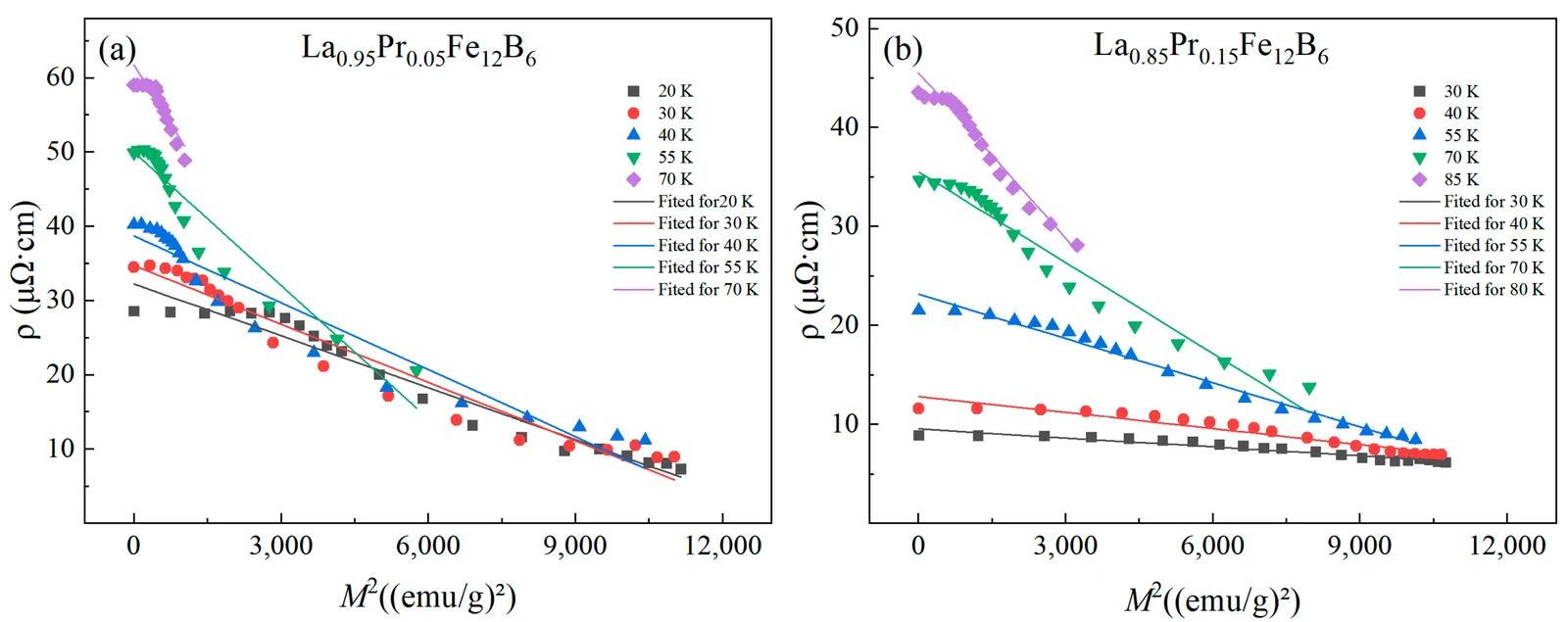
*ρ* versus *M*^2^ isotherms curves of La_0.95_Pr_0.05_Fe_12_B_6_ and La_0.85_Pr_0.15_Fe_12_B_6_ at different temperatures. (**a**) La_0.95_Pr_0.05_Fe_12_B_6_; (**b**) La_0.85_Pr_0.15_Fe_12_B_6_.

Figure 7 shows the isothermal resistivity *ρ* of La_0.95_Pr_0.05_Fe_12_B_6_ and La_0.85_Pr_0.15_Fe_12_B_6_ plotted against *M*^2^, where *ρ*(*H*) and *M*(*H*) were recorded at temperatures matched within 3 K. Within the de Gennes–Friedel picture of spin-disorder scattering [33], the magnetic contribution to the resistivity scales as 1 − (*M*/*M*_0_)^2^, where *M*_0_ is the saturation magnetization, derived from the magnetization of the sample under a magnetic field varying from 70 to 0 kOe at 3 K. Thus, *ρ* is expected to decrease linearly with *M*^2^. Indeed, for the isotherms near *T*_C_, *ρ* exhibits an approximately linear decrease with increasing *M*^2^, and the linear fits reproduce the experimental data well over most of the magnetization range. The magnitude of the slope |d*ρ*/d*M*^2^| grows markedly as *T* approaches *T*_C_, indicating that the carrier scattering is most sensitive to the magnetization in the region where the field-induced metamagnetic (itinerant-electron metamagnetic, IEM) transition takes place. For *x =* 0.05, the 20 K isotherm shows an initial plateau at low *M*^2^ followed by a steep drop, reflecting the antiferromagnetic state at low fields and the subsequent field-induced transition to the ferromagnetic state; similarly, deviations from the linear behavior are visible for the isotherms well above *T*_C_ (70 K for *x =* 0.05 and 85 K for *x =* 0.15). These observations, together with the estimate that an orbital Lorentz-force contribution would amount to only ~10^−4^, rule out a significant orbital contribution and identify the suppression of spin-disorder scattering, driven by the field-induced IEM transition, as the dominant origin of the large negative magnetoresistance.

## 4. Discussion

The experimental results presented above reveal a strong coupling between the magnetic phase transitions and electrical transport behavior in La_1−*x*_Pr*_x_*Fe_12_B_6_ compounds. In the following subsections, the key findings are analyzed further and compared with the literature to deepen the understanding of the underlying transport mechanisms.

### 4.1. Origin of the Transport Response and Relaxation Behavior

The transport behavior of these polycrystalline intermetallics can be discussed within Matthiessen’s rule, *ρ*(*T*) = *ρ*_0_ + *ρ*_ph_(*T*) + *ρ*_mag_(*T*), in which *ρ*_0_ is the temperature-independent residual resistivity generated by every deviation from a perfectly periodic lattice, while *ρ*_ph_(*T*) and *ρ*_mag_(*T*) describe the scattering of the conduction electrons by phonons and by magnetic excitations, respectively [30]. Since the residual term is large, we first examine the structural origin of *ρ*_0_, then the temperature-dependent terms carrying the magnetic-transition signature. Three structural sources feed *ρ*_0_ in these samples. First, because the ingots are polycrystalline, grain boundaries scatter the carriers; second, there is interfacial scattering at the Fe_2_B inclusions (9.5 wt.% in La_0.95_Pr_0.05_Fe_12_B_6_ versus 3.8 wt.% in La_0.85_Pr_0.15_Fe_12_B_6_), each inclusion contributing an incoherent interface whose effective cross-section far exceeds its volume fraction; and third, there is point disorder from the random La/Pr occupation. That these defects dominate the transport is borne out by the magnitude of the resistivity itself: the residual resistivity (6.6–20 μΩ·cm) is comparable to the 150 K value (~59 μΩ·cm for La_0.85_Pr_0.15_Fe_12_B_6_), so the phonon term is modest and *ρ*(150 K)/*ρ*(3 K) remains low—the fingerprint of a metal whose mean free path is limited by structure rather than thermal vibrations. This also explains why the more Fe_2_B-rich La_0.95_Pr_0.05_Fe_12_B_6_ is systematically more resistive (Figure 2).

For La_0.85_Pr_0.15_Fe_12_B_6_, the peak of d*ρ*/d*T* near 61 K is very close to the Curie temperature (*T*_C_ = 63 K). This phenomenon can be understood in terms of critical spin scattering at the magnetic phase transition: near the ferromagnetic ordering temperature, spin-density fluctuations reach a maximum, producing the strongest spin-dependent scattering and thus the largest temperature coefficient of resistivity [30]. This observation is consistent with the classical Mott theory of electron transport in ferromagnetic metals and verifies the strong coupling between magnetic order and electrical transport in this system.

The step-like *MR* jumps observed at 3 K (Figure 3, Figure 4 and Figure 5) and their gradual smearing with increasing temperature are directly correlated with the step-like metamagnetic transitions in the magnetization curves [12,13,14]. Microscopic modeling of itinerant-electron metamagnetism reproduces precisely this phenomenology, i.e., a field-driven level crossing between a low-moment and a high-moment state whose free-energy barrier is progressively washed out by thermal fluctuations [34]. Fixed-spin-moment calculations have identified two inequivalent Fe sites together with low- and high-moment states in LaFe_12_B_6_ [10,11], providing a natural microscopic basis for the observed multi-step behavior: each resistivity jump corresponds to the flipping process of a magnetic sublattice. The two-step transition in La_0.95_Pr_0.05_Fe_12_B_6_ indicates two successive metamagnetic processes (AFM–intermediate–FM), while the disappearance of the high-field step near 67 kOe in La_0.85_Pr_0.15_Fe_12_B_6_ suggests that increased Pr content stabilizes the FM arrangement and simplifies the magnetic transition path.

Below the first critical field (~41 kOe at 3 K), the resistivity rises monotonically with field, and the positive *MR* reaches about 20% just before the transition. Classical orbital magnetoresistance, arising from the Lorentz-force deflection of the conduction electrons, is the positive magnetoresistance mechanism common to all metals [23]. However, it becomes appreciable only when the carrier mean free path is long compared with the cyclotron radius, which is not the case in a poor metal with a residual resistivity of 6.6–20 μΩ·cm. The apparent mean free path *l* of the conduction electrons can be estimated from the residual resistivity using the standard Boltzmann relation:

(3) $$\rho =\frac{3\pi^{2}\hbar }{e^{2}k_{F}^{2}l},$$

where ***k***_F_ = (3π^2^ *n*)^1/3^ is the Fermi wave vector and *n* = ***z***/*V*_f.u._ is the conduction-electron density, with ***z*** the number of conduction electrons per formula unit [35]. Using the refined unit-cell volumes of our compounds (*V* = 603.5 Å^3^ and 602.6 Å^3^ for *x* = 0.05 and 0.15, respectively; from Rietveld refinement) over the physically plausible range 1 electron per f.u. up to one conduction electron per atom (i.e., *z* = 1–19, corresponding to *n* = 5 × 10^27^–9.4 × 10^28^ m^−3^), we obtain *l* ≈ 3–22 nm for *x* = 0.05 and 9–66 nm for *x* = 0.15, respectively, one to two orders of magnitude larger than the interatomic spacing, with *k*_F_*l* ≈ 44–350 ≫ 1. Since *ρ*_0_ remains one to two orders of magnitude below the Ioffe–Regel saturation resistivity of transition-metal compounds (~0.1–0.2 mΩ·cm), both samples are well within the weak-disorder metallic regime. The orbital (Lorentz-force) contribution is therefore negligible even at 40 kOe, far smaller than the ~20% measured, ruling out an orbital origin for the positive *MR*.

A more plausible origin lies in the fragile magnetic ground state of this family: the AFM phase is an amplitude-modulated incommensurate structure with a propagation vector ***k*** = (1/4, 1/4, 1/4) and a very small ordered Fe moment of only ≈0.43 μB [12]. Below the critical field, a moderate external field progressively distorts this weakly ordered spin arrangement, transiently enhancing spin-disorder scattering and thereby raising the resistivity. Since scattering in the FM spin structure is weaker than in the AFM one, this positive contribution remains confined to the narrow field window below the metamagnetic transition. Once the metamagnetic transition is triggered, the suppression of spin-flip scattering dominates over this low-field spin-disorder term, leading to a sharp drop in resistivity and the emergence of giant negative *MR*.

The increase in maximum *MR* magnitude with temperature in the low-temperature region (−59% at 3 K to −75% at 15 K for *x* = 0.05) reflects the weakening of the AFM exchange interaction by thermal fluctuations. At very low temperatures, the AFM state is highly stable and the field-induced transition remains incomplete; as temperature rises, the magnetic structure becomes more sensitive to the external field, and the *MR* ratio gradually increases. The maximum *MR* value first increases and then decreases with temperature, with the peak located near the magnetic phase transition temperature. This shift closely tracks the increase in *T*_C_ induced by Pr doping. A pronounced negative *MR* persists above *T*_C_ (−72% at 55 K for *x* = 0.05, that is, about 8 K above its Curie temperature), which indicates that short-range magnetic order still survives in the paramagnetic region. The external field aligns these short-range ordered moments, reducing spin disorder scattering. This behavior is characteristic of itinerant-electron magnets with strong exchange interactions and differs from the weak paramagnetic *MR* of ordinary metals [9,34].

The magnetic hysteresis and demagnetization relaxation observed in this study arise from two coupled mechanisms: (1) impurity phases and structural defects act as pinning centers for domain walls and phase boundaries; (2) kinetic arrest of the first-order phase transition [36], a scenario first established for the Gd_5_(Si, Ge)_4_ family [5,6] and subsequently invoked for LaFe_12_B_6_ [9], whereby at low temperatures, the thermal activation energy is insufficient to overcome the phase-transition energy barrier, and the field-induced FM phase cannot relax back to the equilibrium AFM state upon field removal. As temperature increases, thermal activation enhances relaxation and the hysteresis gradually vanishes, consistent with the near-complete overlap of magnetization and demagnetization curves observed at 120 K.

### 4.2. Comparison with Related Magnetoresistance Systems

The *MR* effect in La_1−*x*_Pr*_x_*Fe_12_B_6_ differs from other magnetoresistance systems. Unlike the colossal magnetoresistance (CMR) in perovskite manganites, which is driven by charge-order melting and the double-exchange mechanism [1], the giant negative *MR* in the present system originates predominantly from the field-induced magnetic structure transition, without the involvement of charge or orbital ordering. In sputtered NiFe/Cu/NiFe planar GMR structures, the field-induced resistance change is only ~10^−3^ and strongly geometry-dependent [37], whereas the bulk compounds studied here, without any multilayer engineering, show a response two orders of magnitude larger. However, the present system’s application as a continuous magnetic field detector is limited owing to its significant magnetic hysteresis and low sensitivity. Furthermore, the *MR* effect and the magnetocaloric effect in this material share the same magnetic phase transition, suggesting the possibility of integrating magnetic refrigeration with a field-triggered resistive-switching function at cryogenic temperatures, which holds promise for applications in cryogenic engineering and space technology [4,38].

The low-temperature irreversibility observed here is the transport signature of a metastable, kinetically arrested state. A comparable behavior has been reported for the Ce-substituted compound, where the magnetoresistance remains constant at fixed temperature and field for an incubation time of several hundred seconds before collapsing [30]. Such a spontaneous jump is the signature of an explosive instability of the arrested AFM matrix, and it closely resembles the isothermal martensitic transformation of Fe-Ni alloys [36]. Two consequences follow for La_1−*x*_Pr*_x_*Fe_12_B_6_. First, the incubation time is not an intrinsic constant, since it changes by nearly an order of magnitude between resistive and magnetic holding, reflecting the nucleation statistics of FM rather than a material relaxation time. Second, the pronounced hysteresis and the incomplete recovery at 3 K (Figure 3, Figure 4 and Figure 6) represent the low-temperature expression of the same kinetic arrest, so that spontaneous, time-dependent resistivity jumps are expected in La_1−*x*_Pr*_x_*Fe_12_B_6_ near its critical fields (~41 and ~67 kOe for *x* = 0.05). Isothermal resistivity-relaxation measurements performed in small field steps (1 kOe) around these fields, with a temperature stability better than 10 mK, would provide the decisive test.

In the present compounds, the resistivity drop is dominated by the suppression of spin-dependent scattering upon the field-induced alignment of the Fe moments, while the step-like character of the curves reflects the first-order, avalanche-like nature of the underlying transition. This interpretation is consistent with the negative *MR* recently reported for the ferrimagnetic half-Heusler alloy Co_0.5_Mn_1.5_Al, where the field-induced suppression of spin-disorder and magnon scattering was likewise identified as the dominant mechanism [39]. The *MR* evolves smoothly with temperature and field, whereas the abrupt, step-like response observed here directly mirrors the first-order, avalanche-like metamagnetic transition. La_0.95_Pr_0.05_Fe_12_B_6_ combines the largest *MR* measured (−78% at 40 K) with a two-step transformation at 3 K, whereas the higher Pr content shifts the *MR* maximum to a higher temperature (−68% at 70 K) at the expense of its magnitude. This trade-off between the magnitude of the *MR* and the temperature at which it is maximized is the transport analogue of the doping-induced shift of the magnetic phase-transition temperature reported previously [28], and it provides an additional degree of freedom for tailoring these materials to a given cryogenic operating temperature. In the hydrogen-liquefaction window (20–77 K), the present compounds retain a reversible magnetocaloric response of −Δ*S*_M_^max^ = 9.4 and 8.8 J/(kg·K) (0–5 T, *x* = 0.05 and *x* = 0.15) [28], comparable to representative 40–80 K refrigerants such as DyCoNi (9.87 J/(kg·K) at 67 K) and Gd(Cu_0.88_Ni_0.12_)_2_ (10.3 J/(kg·K) at 60 K), while using lighter, lower-cost rare earths than the heavy-*RE* Laves phases DyAl_2_ and HoCo_2_ [40,41,42,43]. Recent work on the parent compound has revealed giant magnetostriction and colossal negative thermal expansion accompanying the same metamagnetic transition [18]. In La_1−*x*_Pr*_x_*Fe_12_B_6_, this strong coupling between spin, lattice and charge degrees of freedom is directly reflected in the transport properties, whose extreme sensitivity to the magnetic state, also manifested under hydrostatic pressure [15], underlies the giant negative *MR* reported here.

## 5. Conclusions

Combining magnetic and electrical transport measurements of La_1−*x*_Pr*_x_*Fe_12_B_6_ (*x* = 0.05, 0.15), we establish how rare-earth substitution governs the magneto-transport response of this itinerant-electron metamagnet. The main conclusions are as follows:

(1) The electrical transport of La_1−*x*_Pr*_x_*Fe_12_B_6_ is governed by its magnetic state. Both compounds are metallic, yet their residual resistivity (6.6–20 μΩ·cm) is set by the Fe_2_B impurity content (9.5 and 3.8 wt.% for *x* = 0.05 and 0.15), and d*ρ*/dT of La_0.85_Pr_0.15_Fe_12_B_6_ peaks at 61 K, essentially at its Curie temperature of 63 K, that is, at the onset of critical spin scattering. Resistivity therefore acts as a sensitive probe of the magnetic phase transition in this family: the field-aligned FM state is the highly conducting state, whereas far above *T*_C_ (e.g., ≥85 K for *x* = 0.05), the resistivity of the paramagnetic state is almost unaffected by the field.

(2) Magnetoresistance reproduces the magnetization process step by step. At 3 K, La_0.95_Pr_0.05_Fe_12_B_6_ shows two abrupt resistivity drops, at 41 and 67 kOe, that coincide with the material’s two metamagnetic jumps, whereas La_0.85_Pr_0.15_Fe_12_B_6_ exhibits only the 41 kOe step; the vanishing of the high-field step indicates that a higher Pr content stabilizes the ferromagnetic arrangement and removes the intermediate magnetic state. Each drop is preceded by a positive *MR* of about +20% for *x =* 0.05 but of only a few percent for *x =* 0.15, which can be attributed to field-induced distortion of the weakly ordered AFM.

(3) The giant negative magnetoresistance is large, compositionally tunable, and persists above *T*_C_. The largest *MR* values measured are −78% at 40 K for *x =* 0.05 and −68% at 70 K for *x =* 0.15, following the same trend as the Pr-induced shift of the transition temperature from 47 to 63 K, and a value of −72% is still found at 55 K, about 8 K above *T*_C_ for *x =* 0.05, before the effect collapses to −7% at 85 K and −1% at 100 K. This persistence above *T*_C_ demonstrates that the dominant scattering contrast is that between the field-aligned ferromagnetic state and the short-range-ordered paramagnetic state, rather than that between the AFM and FM states.

(4) The low-temperature field-induced transformation is kinetically arrested and only partially reversible. Upon field removal, the resistivity fails to return to its initial value over a broad low-temperature range—remaining strongly arrested for *x =* 0.05 up to about 40 K, and persisting, though gradually weakening, up to roughly 85 K for *x =* 0.15—whereas at 120 K, the field-increasing and field-decreasing curves coincide for both compounds. This behavior is the signature of a martensitic-like kinetic arrest, in which impurity pinning and the first-order free-energy barrier jointly hinder the relaxation of the field-induced ferromagnetic phase, and it implies that the virgin antiferromagnetic state can be restored only by warming the sample well above *T*_C_ and cooling it again in zero field.

In summary, the charge transport in La_1−*x*_Pr*_x_*Fe_12_B_6_ acts as a direct probe of the field-induced metamagnetic transition: the transformation generates a giant negative *MR* of up to −78%, whose maximum can be tuned by adjusting the Pr content, and the crossover from reversible to kinetically arrested behavior defines the usable window of the field-induced state.

## Figures and Tables

**Table 1:** Rietveld refinement parameters and impurity content of La_0.95_Pr_0.05_Fe_12_B_6_ and La_0.85_Pr_0.15_Fe_12_B_6_ compounds.

Parameter	La_0.95_Pr_0.05_Fe_12_B_6_	La_0.85_Pr_0.15_Fe_12_B_6_
a (Å)	9.587	9.584
c (Å)	7.581	7.576
*R*_wp_ (%)	4.079	3.885
*R*_B_ (%)	3.174	3.028
Fe_2_B (wt.%)	9.5	3.8

## Data Availability

The original contributions presented in this study are included in this article. Further inquiries can be directed to the corresponding author.
